# Decoupling the Evolution of the Light-Absorption Properties of Primary and Secondary Organic Aerosol Produced from Duff Burning

**DOI:** 10.1021/acsestair.5c00274

**Published:** 2025-11-21

**Authors:** Muhammad I. Abdurrahman, Chase K. Glenn, Robert Penland, Ryan P. Poland, Jonathan H. Choi, Elijah T. Roberts, I. Jonathan Amster, Geoffrey D. Smith, Rawad Saleh

**Affiliations:** † School of Environmental, Civil, Agricultural, and Mechanical Engineering, 1355 University of Georgia, Athens, Georgia 30602, United States; ‡ Department of Chemistry, University of Georgia, Athens, Georgia 30602, United States; § 53777 Aerodyne Research Inc., Billerica, Massachusetts 01821, United States

**Keywords:** secondary organic aerosol, primary organic aerosol, biomass burning, brown carbon, photochemical aging, duff combustion, light absorption

## Abstract

This study investigated the coevolution of the light-absorption properties of biomass-burning primary and secondary organic aerosol (POA and SOA) during photochemical aging. We performed smoldering combustion of duff and photochemically aged the emissions in an oxidation flow reactor (OFR). We retrieved the imaginary part of the refractive index (*k*) of the POA, aged POA (APOA), SOA, and aged OA (AOA), which includes both APOA and SOA. Photochemical aging induced competing effects on AOA absorption: (1) slight photoenhancement in POA, and (2) formation of very weakly absorbing SOA, with midvisible *k* an order of magnitude smaller than that of the POA, that photobleached rapidly with further oxidation. The latter effect dominated, resulting in a net decrease in AOA absorption. Changes in chemical composition corroborated the evolution in light-absorption properties. While POA exhibited minimal change in chemical composition due to photochemical aging, SOA underwent significant chemical transformation consistent with the observed photobleaching. We also demonstrated that the previously used indirect method, which estimates SOA absorption by subtracting fresh POA absorption from AOA absorption, can lead to severe overestimation of SOA absorption. Our findings underscore the importance of considering the distinct optical evolution of SOA and POA during photochemical aging.

## Graphical abstract

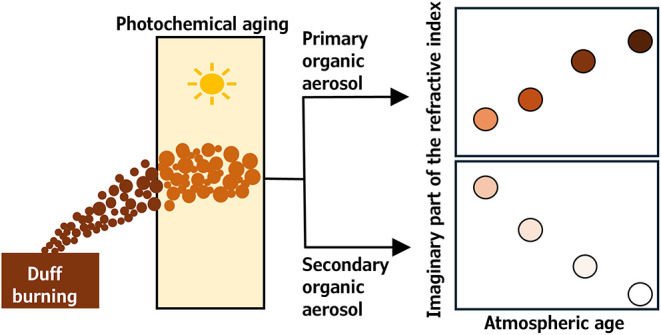

## 1 Introduction

The light-absorption properties of organic aerosol (OA) in biomass-burning emissions remain among the most significant uncertainties in radiative-transfer calculations.^1–4^ While black carbon (BC) is the most potent particulate light absorber, a significant fraction of atmospheric light absorption, particularly in the near-ultraviolet (UV) and visible wavelengths, is attributed to light-absorbing OA, or brown carbon (BrC).^5–12^ In addition to primary organic aerosol (POA) that is emitted directly during combustion, the chemical processing of biomass-burning smoke in the atmosphere leads to the formation of secondary organic aerosol (SOA), often in amounts comparable to POA.^13–19^ There is evidence that biomass-burning SOA exhibits appreciable light absorption and is classified as BrC.^4,9,20–23^ Nevertheless, despite growing recognition of its significance, the light-absorption properties of biomass-burning SOA are still poorly constrained.^4,21,24^

Multiple studies have examined the light absorption of SOA formed from surrogate biomass-burning precursors such as phenols, furans, and aromatic compounds.^25–37^ For example, SOA from nitrophenol and methoxyphenol oxidation has shown appreciable near-UV absorption,^32,33,38^ while even larger absorption has been reported for these same precursors under high-NO*_x_* photooxidation conditions.^29,30,37–39^ Although these findings demonstrate that biomass-burning SOA can absorb light, the light-absorption properties inferred from surrogate species may inadequately represent those of SOA in actual biomass-burning emissions due to the complexity of precursors in actual combustion emissions.^38^

Previous studies that reported the light-absorption properties of SOA from actual biomass-burning emissions,^9,40^ inferred them from absorption measurements of aged OA (AOA), which is a mixture of both SOA and POA. Those studies retrieved SOA light-absorption properties by subtracting POA contribution to absorption from the AOA absorption, assuming that POA absorption remained unchanged during photochemical aging.^9,40^ A potential drawback of this approach is that it does not account for the possible chemical transformation of the POA constituents during aging, which may alter its absorption and thus bias the inferred SOA light-absorption properties. This highlights the need to measure the optical properties of SOA directly to avoid misattribution and improve the accuracy of the estimates of SOA light-absorption properties.

Moreover, SOA is chemically distinct from POA and is expected to follow a different chemical transformation pathway during atmospheric aging,^38,41,42^ with important implications for the evolution of their respective light-absorption properties. While previous studies have investigated the photochemical evolution of biomass-burning OA over extended aging time scales,^38,43–49^ most have focused on AOA without distinguishing between POA and SOA, thus confounding the interpretation of their respective contributions to changes in absorption. Although POA aging has been studied separately, often using denuders to remove vapors and suppress SOA formation, with outcomes ranging from net photobleaching^38,50,51^ to net photoenhancement,^22^ the independent evolution of SOA and its contribution to absorption changes in the AOA remain unresolved. Given the complexity of overlapping processes during photochemical aging, such as POA chromophore transformation, SOA formation (absorbing or nonabsorbing), and transformation of SOA chromophores, decoupling the optical evolution of POA and SOA is essential for accurately interpreting changes in AOA absorption.

Two critical knowledge gaps thus emerge. First, the light-absorption properties of SOA in actual biomass-burning emissions have not been measured directly. Second, there are no studies that have tracked the coevolution of the light-absorption properties of POA and SOA during photochemical aging of biomass-burning emissions. In this study, we address these gaps by conducting controlled photochemical-aging experiments of biomass-burning emissions derived from the smoldering of duff. We alternatively isolated POA and SOA and oxidized each separately at different levels of OH exposure in an oxidation flow reactor (OFR). POA was isolated by removing vapors from the emissions, while SOA was isolated by removing primary particles. We quantified changes in light-absorption properties as a function of OH exposure and performed chemical analysis to evaluate compositional changes of the OA components.

## 2 Methods

### 2.1 Overview of the Experiments

As illustrated in Figure 1, we conducted smoldering combustion of duff in a 7.5 m^3^ Teflon chamber, conditioned at a relative humidity of 15%. Duff, a partially decomposed organic layer that forms in forest floors that have undergone long periods of fire exclusion,^52,53^ was collected from Whitehall Forest in Athens, GA, and is representative of duff in the Piedmont ecoregion in the Southeastern U.S. Duff typically becomes available for combustion (i.e., ignites) when it is dry during drought-induced wildfires.^52–54^ Therefore, the duff was dried in an oven at 65 °C to reduce the moisture content to less than 5%. Furthermore, due to its compactness and relatively low carbon content, duff predominantly exhibits low-temperature smoldering combustion^52,55^ and primarily emits OA, with small amounts of BC.^7^ Each burn involved the combustion of 10 g of duff, which smoldered for 5–7 min. A centrally mounted fan promoted mixing in the chamber, and sampling started 20 min after the conclusion of the burn. The chamber was effectively sealed and operated without makeup air during each experiment. The emissions were oxidized with OH radicals in an Aerodyne oxidation flow reactor (OFR).^56^ The operation of the OFR is described in detail in Section 2.2.

**Figure 1:**
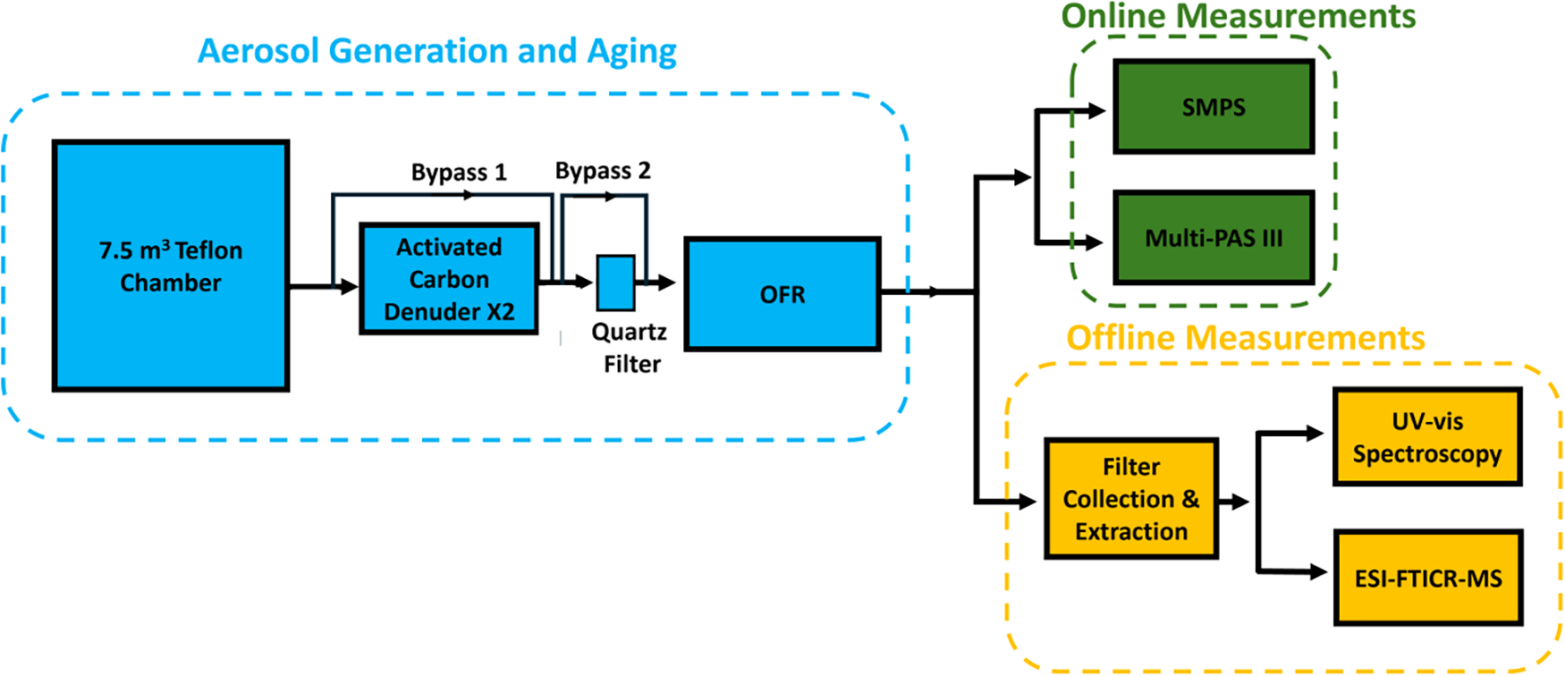
Schematic of the experimental setup.

As summarized in Table 1, the experiments featured four configurations designed to isolate and characterize the light-absorption properties of POA, aged OA (AOA–includes both aged POA and SOA), aged POA (APOA), and SOA:

(1) All emissions, including particles and vapors, were directed from the chamber into the OFR. The OFR UV lights were kept off to measure the light-absorption properties and size distribution of freshly emitted POA.
(2) Same as (1), but UV lights were turned on to initiate photochemical aging, exposing the smoke (particles and vapors) to OH radicals and generating SOA. The resulting aerosol, AOA, consisted of a mixture of APOA and SOA.
(3) Emissions were passed through two activated carbon denuders to remove vapors before entering the OFR to isolate the effect of aging on POA alone. This configuration effectively removed SOA precursors and suppressed SOA formation, allowing only POA to undergo photochemical aging. The resulting aerosol was APOA. Similar denuder-based approaches for isolating POA have been successfully applied in previous OFR studies.^22,38,50,51^

(4) Emissions were passed through a quartz filter to remove particles, allowing only vapors to enter the OFR. Upon photochemical aging, the formation of new particles was attributed solely to SOA produced from gas-phase precursors.

**Table 1:** Summary of the Four Experimental Configurations Used to Isolate and Characterize the Light-Absorption Properties of Biomass-Burning OA Components

OA type	description	condition
(1) POA	fresh OA emissions directly from smoldering combustion	UV lights OFF, denuders bypassed, quartz filter bypassed
(2) AOA	photochemically aged mixture of APOA and SOA	UV lights ON, denuders bypassed, quartz filter bypassed
(3) APOA	aged POA particles with SOA formation suppressed	UV lights ON, denuders ON, quartz filter bypassed
(4) SOA	SOA formed from the oxidation of gas-phase precursors in the absence of primary particles	UV lights ON, denuders bypassed, quartz filter ON

Online measurements of aerosol size distributions were performed using a Scanning Mobility Particle Sizer (SMPS, TSI 3882) in the size range from 10 to 500 nm. Corrections for diffusion losses and multiple charges were applied using the built-in settings provided on the SMPS software (AIM, TSI 10.3.1). The measured size distributions were integrated to yield the total aerosol mass concentrations, assuming a particle density of 1.2 g/cm^3^, reported for OA.^57^ We note that BC constituted less than 0.5% of the aerosol mass (see Tables S1 and S2 of the Supporting Information (SI)) and was thus not accounted for in calculating the mass concentrations. Aerosol absorption coefficients (*b*_abs_, Mm^–1^) at three different wavelengths (422, 532, and 782 nm) were measured using a custom-built Photoacoustic Spectrophotometer (Multi-PAS III).^58^ Because of the small OA absorption at 782 nm, only *b*_abs_ at 422 and 532 nm were used in the analysis.

### 2.2 Operation of the Oxidation Flow Reactor

The duff-burning emissions were oxidized with OH radicals in the OFR for each of the configurations that involved photochemical oxidation (configurations 2–4), as summarized in Table 1. The OFR consists of a 13 L cylindrical flow chamber equipped with low-pressure mercury lamps emitting UV radiation at 185 and 254 nm wavelengths.

OH exposure within the OFR is influenced by residence time, UV light intensity, water vapor concentration, and OH reactivity (the rate at which the reactive species in the OFR scavenge OH radicals). The OFR was operated at a constant water vapor concentration (RH = 15% at 25 °C) and a constant airflow rate of 7.3 L/min, resulting in an average residence time of approximately 107 s. We varied the OH exposure in our experiments by adjusting the voltage of the UV lights between 1.7 and 2.0 V. Based on the parametrization of Peng et al.,^59^ the estimated OH exposures in our experiments ranged from approximately 6.5 × 10^10^ to 2.6 × 10^11^ molecules cm^–3^ s, corresponding to 0.5 days to 2 days of equivalent atmospheric photochemical aging, assuming an atmospheric OH concentration of 1.5 × 10^6^ molecules cm^–3^.^60–62^

We note that our experiments did not employ gas-phase measurements to quantify the consumption of VOC tracers in the OFR. The reported OH exposure values were estimated from an offline calibration using the PAM OFR software.^59^ Several OFR studies have shown a correlation between OH exposures derived from gas-phase tracer measurements and those estimated using the OFR internal calibration.^41,59,63–65^ The OFR internal-calibration approach, however, does not account for the suppression of OH due to external OH reactivity. Therefore, the OH exposures in our experiment should be considered upper limits. While the actual OH exposure values may be lower than those reported in our experiments, the trends observed due to varying OH exposures (by varying the UV light intensity) in the OFR and the conclusions based on the relative trends are valid because they do not depend on the absolute values of OH exposures.

While chemical transformation in the OFR is predominantly dictated by reactions with OH radicals, it may also result from reactions with ozone and direct photolysis due to UV light exposure within the OFR. The analysis in this study accounts for the combination of these photoinitiated effects, but does not distinguish between the individual effects. These combined effects can lead to a net decrease in the OA light absorption due to the degradation or loss of chromophores (commonly referred to as photobleaching)^4^ or a net increase in light absorption due to the formation or transformation of chromophores (commonly referred to as photoenhancement).^4^ We note that these changes in light absorption are not only due to direct interaction with light, and should strictly speaking be referred to as photoinitiated bleaching and photoinitiated enhancement. However, to keep consistent with the literature, we use the terms photobleaching and photoenhancement with the understanding that they refer to photochemical transformations in general.

### 2.3 Retrieval of Light-Absorption Properties

The light-absorption properties of BrC are commonly quantified using the mass absorption cross-section (MAC) and the imaginary part of the refractive index (*k*).^4^ MAC, expressed in units of m^2^ g^–1^, provides a convenient way to quantify aerosol light absorption at a given wavelength (λ) and is often used in conjunction with Absorption Ångström Exponent (AAE) to describe the wavelength dependence of the absorption (MAC ∼ λ^–AAE^). However, while widely used, MAC and AAE are not true intensive properties as they are dependent on particle size.^4^ In comparison, *k*, the imaginary part of the refractive index, is a more fundamental parameter and is independent of the particle size.^23,66^

We retrieved *k* of the duff-burning OA emissions from each experimental configuration (POA, AOA, SOA, and APOA; see Table 1) using the optical closure approach described in our previous studies.^5,7,57^ This approach involves fitting *b*_abs_ obtained from Mie calculations to the *b*_abs_ measured using the Multi-PAS III (Section 2.1) with *k* as a free parameter. BC contribution to the total absorption was accounted for in the optical closure analysis, assuming external mixing between BC and OA particles.^7^ BC absorption coefficient (*b*_abs,BC_) was calculated using Mie theory, with a BC refractive index of *m* = 1.85 + 0.71*i*.^66^ We assumed that the shape of the BC size distribution is the same as that of the overall aerosol measured using the SMPS (Section 2.1) and was scaled to the ratio of elemental carbon (EC) to organic matter (OM) in the aerosol. The mass concentrations of EC and OM (see Tables S1 and S2 in the Supporting Information (SI)) were measured using an organic carbon (OC) and EC (OCEC) analyzer (Sunset Laboratory, Model 5 L) following the Niosh-870 protocol.^67^ This allowed us to isolate OA absorption (*b*_abs,OA_) from the total measured absorption. Mie calculations were then performed assuming homogeneous spherical OA particles with a real part of the refractive index of 1.55.^66^ We retrieved *k* values of OA at 532 and 422 nm and also represented the wavelength-dependent *k* using *k* of OA at 550 nm (*k*_OA,550_) and the wavelength dependence (*w*), assuming power-law wavelength dependence (eq 1) in order to categorize the OA into BrC optical classes.^4^ For context, readers more familiar with AAE can note that AAE ≈ *w* + 1 for particles much smaller than the wavelength of absorption.

1 $$k_{\mathrm{OA},\lambda }=k_{\mathrm{OA},550}{\left(\frac{550}{\lambda }\right)}^{w}$$

In addition to retrieving the *k* for the isolated SOA configuration directly (*k*_SOA_), we also estimated *k*_SOA_ using an indirect method analogous to that employed in previous studies, Saleh et al.^9^ and Kumar et al.^40^ These studies inferred SOA absorption by subtracting the contribution of POA from the absorption of AOA, assuming that the measured absorption of AOA is a mass-weighted average of the absorptions from POA and SOA. In this study, we applied this approach using two scenarios:

(1) Assuming POA absorption remained unchanged with aging, in which case we used the *k* value of POA (*k*_POA_) to estimate *k*_SOA_ using
2 $$k_{\mathrm{SOA}}=\frac{k_{\mathrm{AOA}}-{(m_{\mathrm{POA}}\times k}_{\mathrm{POA}})}{{(1-m}_{\mathrm{POA}})}$$
  Where *m*_POA_ is the mass fraction of POA in the AOA.
(2) Accounting for the chemical evolution of POA by using the *k* value of APOA (*k*_APOA_), thus, *k*_SOA_ was estimated using
3 $$k_{\mathrm{SOA}}=\frac{k_{\mathrm{AOA}}-{(m_{\mathrm{POA}}\times k}_{\mathrm{APOA}})}{{(1-m}_{\mathrm{POA}})}$$
  To facilitate the discussion, we will hereafter refer to *k*_SOA_ predicted using this indirect approach as *k*_SOA_ (from POA) and *k*_SOA_ (from APOA).

We also retrieved *k* of the methanol-soluble fractions of the POA, APOA, SOA, and AOA from light-absorption measurements using a UV–vis spectrometer following the procedure described by Atwi et al.^5^ For each experiment, we collected aerosol samples on 47 mm Teflon filters (0.45 μm pore size, Whatman) at a flow rate of 5 LPM, targeting a mass loading of ∼300 μg. The filters were then inserted in vials containing 2 mL of methanol and sonicated for 20 min. Further sonication has been shown to have no effects on extraction efficiency.^5^ The extracts were subsequently filtered using a glass syringe with a metal luer-lock housing a 13 mm Teflon filter (0.2 μm pore size, Sterlitech Corporation, PTU021350) to remove any residual insoluble material that was mechanically dislodged during sonication. Blank filters were extracted similarly and used for background correction. We measured the absorbance of the extracted solution using a UV–vis spectrometer (Agilent, Cary 60) across a wavelength range of 200 nm–800 nm at a resolution of 1 nm. We then used the measured absorbance to calculate the absorption coefficient, α, and *k* of the methanol-soluble OA

4 $$\alpha =\frac{\ln\left(10\right)A\left(\lambda \right)}{C\times L}$$

5 $$k\left(\lambda \right)=\frac{\lambda \times \alpha \times \rho }{4\times \pi }$$

Where *A* represents the absorbance of the extracts, λ is the wavelength, ρ is the density of OA assumed to be 1.2 g/cm^3^,^5^ *L* (1 cm) is the optical path length, and *C* is the mass concentration of the OA molecules in the solution. To quantify *C*, we deposited 200 μL of each sample extract onto a prebaked quartz filter punch (1.5 cm^2^), dried it under a stream of ultrapure nitrogen, and quantified the OC mass using the OCEC. This OC mass was then converted to mass of organic matter (OM) using an assumed OM/OC ratio of 1.8.^5^ These calculations are detailed in the SI. Finally, we obtained *k*_550_ and *w* of the methanol-soluble OA by fitting a power-law function (eq 1) to the wavelength-dependent *k* values obtained from eq 5.

### 2.4 Chemical Speciation of OA Molecules

The methanol-extracted solutions were analyzed by electrospray ionization Fourier-transform ion cyclotron resonance mass spectrometry (ESI-FTICR-MS). ESI has been commonly used for analyzing the molecular composition of biomass-burning OA due to its ability to ionize these relatively polar molecules effectively.^7,12,68,69^ The analysis was conducted using a Bruker SolariX XR 12 T FT-ICR mass spectrometer in negative ionization mode, covering a mass-to-charge (*m*/*z*) range of 70–1000. The instrument also used a transient length of 1.667 s, yielding a resolution of ∼430,000 at 400 *m*/*z*.

Each sample was analyzed in triplicate, with each spectrum consisting of an average of 48 scans. The resulting spectra were background-corrected by subtracting the spectrum of blank samples using Bruker Data Analysis software before being processed with MFassignR^70^ for molecular assignments. Initially, peaks were assigned, assuming molecules only comprised carbon (C), hydrogen (H), and oxygen (O). ^13^C isotopes were then filtered to retain only monoisotopic peaks, which underwent subsequent internal mass recalibration before a final molecular assignment. The final molecular assignment included compounds containing C, H, O, and nitrogen (N), enabling the evaluation of O/C and H/C ratios as well as the aromaticity index of the OA molecules.

## 3 Results and Discussion

### 3.1 Evolution of Organic Aerosol Mass Concentration in the Different Experimental Configurations

Figure 2 presents the time series of OA mass concentration for the three photochemical-aging configurations: AOA, APOA, and SOA. For clarity, we only show the time series for OH exposure corresponding to 0.5 days of equivalent atmospheric photochemical aging. Figure 2a illustrates the AOA experimental timeseries. At *t* < 0, POA was measured by keeping OFR UV lights off. The POA mass concentration decays over time due to particle wall losses in the Teflon chamber. Upon turning on the UV lights (*t* = 0), the OA concentration increased relative to the POA baseline, indicating SOA formation. To quantify the enhancement in OA concentration, we fitted an exponential decay function to the POA concentration, which provided an estimate of what the POA concentrations would have been in the absence of photochemical aging at equivalent measurement times. The difference between this fitted POA concentration and the measured AOA concentration provided an estimate of the concentration of newly formed SOA.

**Figure 2:**
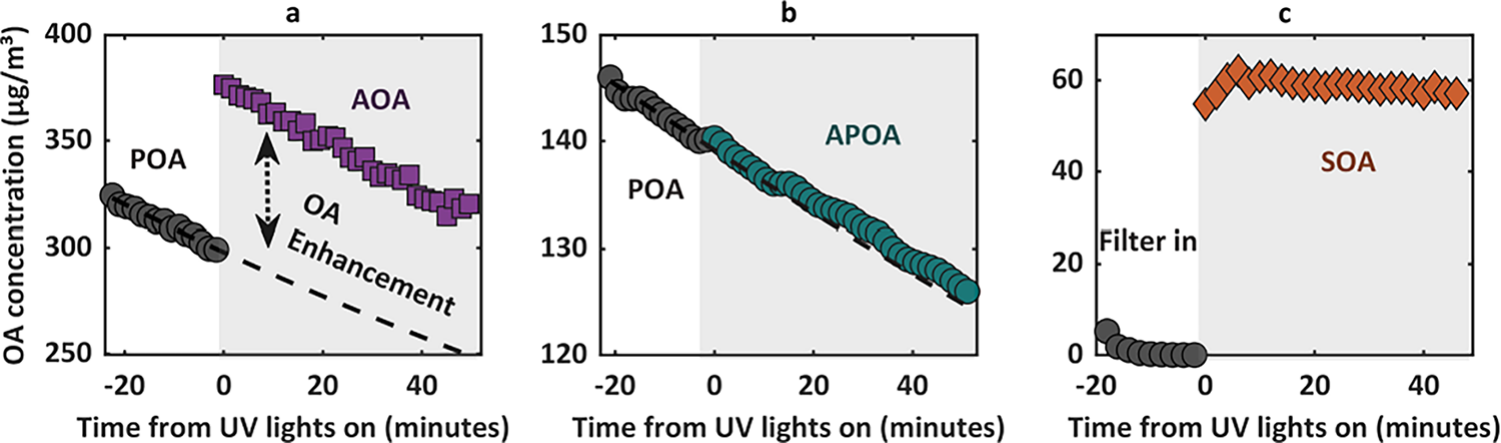
Time series of OA mass concentration for the three photochemical-aging configurations: (a) AOA, (b) APOA, and (c) SOA. The shaded regions indicate periods when the UV lights were on. The black dashed lines in panels a and b represent exponential decay fits to the POA concentration and were used to estimate the mass concentration of the SOA. Transient periods (when the OFR UV lights were turned on) were removed for clarity. Data for AOA, APOA, and SOA are for OH exposure corresponding to 0.5 days of equivalent atmospheric photochemical aging.

The OA enhancement, i.e., the increase in OA mass concentration due to SOA formation, was approximately 25%, 37%, 49%, and 43% for equivalent atmospheric photochemical ages of 0.5 days, 1 day, 1.5 days, and 2 days, respectively. The trend of OA enhancement indicates that during photochemical aging, the total OA concentration initially increases with OH exposure, reaching a maximum at approximately 1.5 days of equivalent aging time, after which it begins to decline. This is consistent with previous observations of increase in aerosol mass due to net functionalization during initial photochemical aging times, followed by net aerosol mass loss due to fragmentation and volatilization at longer aging times.^71–73^

In contrast, the APOA experiment (Figure 2b) showed no appreciable increase in OA concentration after turning the OFR UV lights on. Even though we did not employ gas-phase measurements to quantify SOA precursors, the fact that OA concentrations did not increase in the OFR confirms minimal SOA formation due to the efficient removal of vapors (SOA precursors) from the emissions using activated carbon denuders. Therefore, this configuration effectively isolated the effect of photochemical aging on the POA.

Figure 2c illustrates the SOA experiment, in which POA particles were removed using a quartz filter, and only the vapors were allowed to pass through the OFR. Upon turning the UV lights on, the SOA concentration increased rapidly and remained relatively constant throughout the aging period. This stability highlights that vapor wall-losses in the Teflon chamber were substantially less significant compared to particle wall-losses, contrasting with previous studies that reported substantial vapor wall-losses in Teflon chambers.^74–76^

### 3.2 Evolution of the Light-Absorption Properties of Organic Aerosol Due to Photochemical Aging

As described in Section 2.3, we retrieved the imaginary part of the refractive index (*k*) of POA, APOA, SOA, and AOA across the four levels of OH exposures in the OFR, which correspond to 0.5 days, 1 day, 1.5 days, and 2 days of equivalent atmospheric photochemical age. In addition to retrieving *k*_SOA_ directly from the SOA measurements, we also predicted *k*_SOA_ using an indirect approach, testing the two assumptions described in Section 2.3: (1) assuming that POA absorption remains unchanged during aging −*k*_SOA_ (from POA), and (2) accounting for changes in POA absorption due to photochemical aging −*k*_SOA_ (from APOA). As shown in Figure 3, photochemical aging led to an overall reduction in *k*_AOA,532_ and *k*_AOA,422_. The *k*_AOA_ values underwent a consistent decline up to an OH exposure equivalent to the atmospheric photochemical age of 1.5 days, followed by a plateau between 1.5 days and 2 days. This finding is qualitatively consistent with previous laboratory studies and field measurements of the evolution of light-absorption properties of biomass-burning OA, and has been attributed to the loss of BrC chromophores by photobleaching.^45,77–81^ However, examining the effect of photochemical aging on *k* of the AOA components (APOA and SOA) reveals more complex behavior.

**Figure 3:**
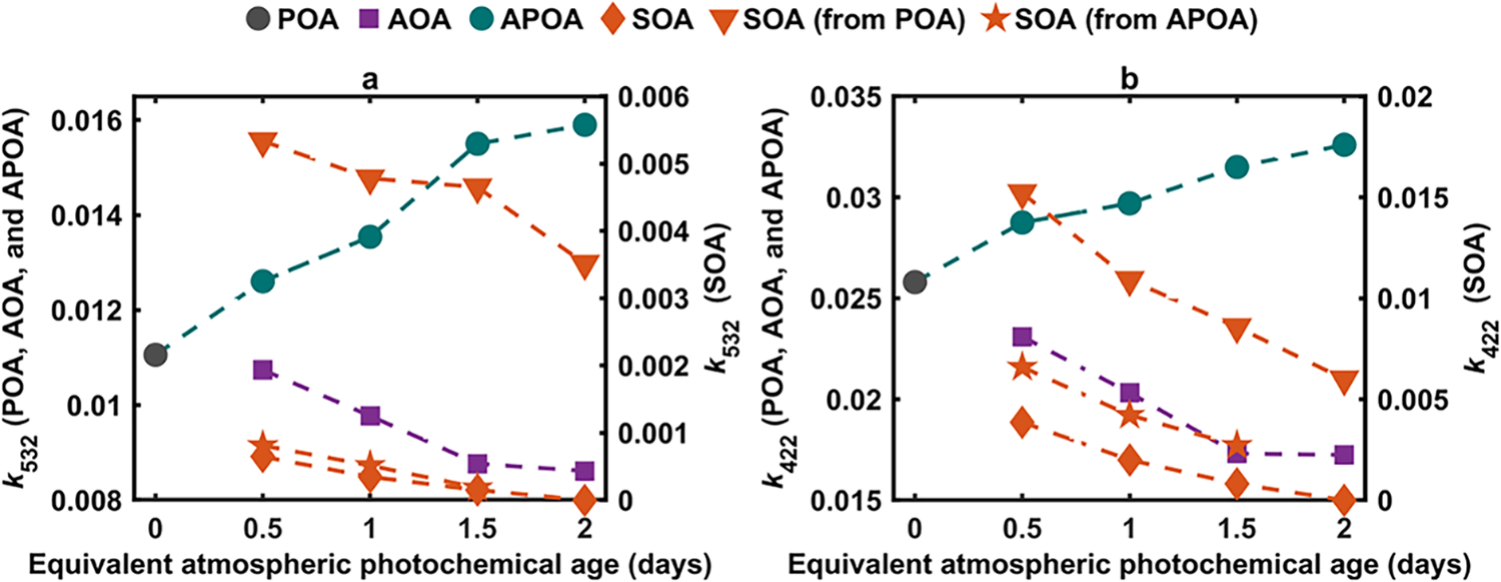
Evolution of the imaginary part of the refractive index (*k*) of OA for the different experimental configurations summarized in Table 1 as a function of equivalent atmospheric photochemical age and for wavelengths of (a) 532 nm and (b) 422 nm. For clarity, values of *k*_SOA_ are shown on a separate (right) *y*-axis. *k*_SOA_ (from POA) and *k*_SOA_ (from APOA) were predicted indirectly as detailed in Section 2.3. Predictions of *k*_SOA_ (from APOA) at 2 days yielded negative values and are not shown.

On the one hand, *k*_APOA_ exhibited an increase over the two-day equivalent atmospheric aging period at both wavelengths, indicating a photoenhancement effect. Similar photoenhancement has been previously reported for the aging of biomass-burning primary BrC^22,38,51,82^ and has been attributed to oxidative processing that transforms BrC chromophores into more conjugated chromophores.^38^ While our results show a progressive increase in the APOA absorption over the two-day aging period, it is possible that this trend may not persist at longer time scales, and absorption may begin to decline as aging progresses further. Sumlin et al.^51^ showed that biomass-burning POA, aged up to 4.5 days, began to undergo photobleaching after ∼3.5 days of equivalent atmospheric aging at 405 and 532 nm.^51^ This suggests that while our moderate aging time scale enhances primary BrC absorption, it is possible that it represents an intermediate phase, with longer-term aging eventually degrading chromophores and reducing absorption.

On the other hand, SOA was significantly less-absorbing than APOA at 0.5 days, with *k*_SOA_ values an order of magnitude smaller than those of *k*_APOA_ at both 532 and 422 nm. Furthermore, *k*_SOA_ decreased sharply with increasing photochemical age, and the SOA was virtually nonabsorbing (*b*_abs_ values were below detection limit) at 2 days. While the decrease in *k*_SOA_ with increased photochemical age can be attributed in part to the formation of less-absorbing or nonabsorbing SOA with continued aging, the virtual disappearance of absorption indicates loss of chromophores due to photobleaching, consistent with previous studies that investigated the photochemical evolution of secondary BrC formed from surrogate precursors.^26,38,81,83–87^

Therefore, photochemical aging led to competing effects on AOA light absorption: (1) increase in absorption of APOA, and (2) the formation of significantly less-absorbing SOA, which lost its absorption with further photochemical aging. Our results indicate that the second effect was more dominant, leading to an overall decrease in AOA light absorption (i.e., decrease in *k*_AOA_). However, the decrease in *k*_AOA_ was not due to photobleaching of primary BrC chromophores, but rather due to mixing with the less-absorbing SOA.

The results in Figure 3 show that predictions of *k*_SOA_ (from POA) (see Section[Sec sec2.3] for details) led to substantial overestimation compared to *k*_SOA_. Specifically, *k*_SOA_ (from POA), obtained using a similar indirect method previously employed to retrieve *k*_SOA_ in biomass-burning emissions,^9,40^ led to an overestimation by approximately a factor of four and eight at 422 and 532 nm, respectively. This drastic overestimation in *k*_SOA_ is explained as follows. POA is significantly more absorbing than SOA. Thus, a small or moderate increase in POA absorption (i.e., *k*_APOA_ > *k*_POA_), if not accounted for, would require a substantial artificial inflation in *k*_SOA_ to yield the same AOA absorption (eq 2). In contrast, *k*_SOA_ (from APOA), which accounted for the increase in *k*_APOA_ due to photochemical aging, yielded much closer estimates to *k*_SOA_ that were within 40% and 20% at 422 and 532 nm, respectively. This finding indicates that assuming constant POA light-absorption properties during aging can lead to inaccurate estimates of SOA light-absorption properties if retrieved indirectly from measurements of POA and AOA. Aging-induced changes in POA light-absorption properties must be accounted for to estimate SOA light-absorption properties using the indirect approach.

### 3.3 Optical Classification

Following Saleh,^4^ Figure 4 categorizes the light-absorption properties for all experimental configurations based on their *k* values at 550 nm (*k*_550_) and the wavelength dependence (*w*) into four optical classes: very weakly absorbing (VW-BrC), weakly absorbing (W-BrC), moderately absorbing (M-BrC), and strongly absorbing (S-BrC). Increasing *k*_550_ and decreasing *w* indicate darker, more absorbing BrC.^4^ For AOA, APOA, and SOA, only *k*_550_ corresponding to 0.5 days of equivalent atmospheric aging are shown in the figure. To contextualize the results, we also include literature values of light-absorption properties of POA from duff burning,^7^ SOA from biomass-burning surrogate precursors,^27,28,30,34,39^ as well as SOA from actual biomass-burning emissions.^9,40^

**Figure 4:**
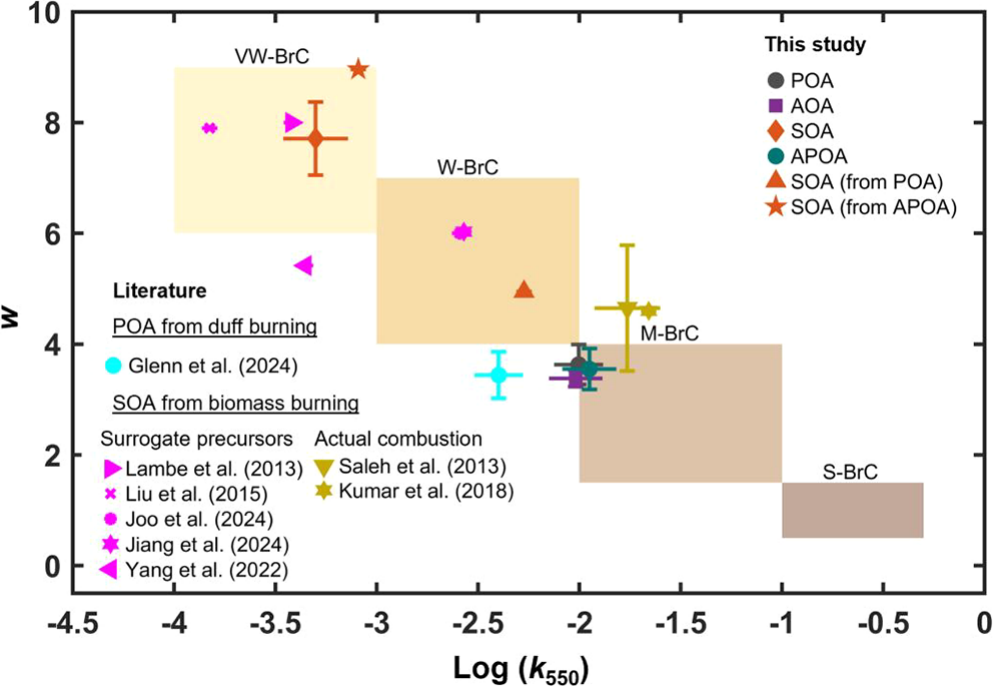
Light-absorption properties of POA, AOA, APOA, and SOA, plotted in log_10_(*k*_550_)–*w* space. Shaded rectangles indicate BrC optical classes proposed by Saleh:^4^ very weakly absorbing (VW-BrC), weakly absorbing (W-BrC), moderately absorbing (M-BrC), and strongly absorbing (S-BrC). Error bars denote standard deviations. For SOA, APOA, and AOA from this study, only values corresponding to 0.5 days of equivalent atmospheric photochemical age are shown. Also shown are literature values for comparison (see text for details). Literature light-absorption properties reported using MAC and AAE were converted to *k*_550_ and *w* using the approach outlined by Saleh.^4^ Numerical values for all data points are presented in Table S3.

As shown in Figure 4, the POA in our experiments falls on the edge between M-BrC and W-BrC, with light-absorption properties comparable to those reported by Glenn et al.^7^ for POA emissions from biomass fuel beds consisting mostly of duff. Differences between our *k*_550_ values and those of Glenn et al.^7^ are likely due to differences in combustion conditions. Nevertheless, the results are consistent in the sense that the low-temperature smoldering combustion characteristic of duff burning emits POA that is less-absorbing than POA emissions from high-temperature combustion.^7^ Furthermore, whereas photochemical aging had a measurable effect on OA light-absorption properties (*k*_AOA_ < *k*_POA_ < *k*_APOA_) as shown in Figure 3, the effect is rather small when considered within the context of the large variability in BrC light-absorption properties,^4^ with POA, AOA, and APOA all falling within the same BrC class (Figure 4).

The measured SOA absorption from this study falls within the VW-BrC class, characterized by a small *k*_550_ (0.0005 ± 0.0001) and a large *w* (7.71 ± 0.66). These values are consistent with previous studies that generated SOA in the laboratory using surrogate biomass-burning precursors, including guaiacol,^28^ toluene,^30^ ethylbenzene,^34^ indole,^39^ and furans.^27^ This reinforces the conclusion that SOA, regardless of its precursor origin or formation conditions, exhibits very weak light absorption and contributes minimally to overall BrC absorption.

Our SOA light-absorption properties are significantly different from those reported by Saleh et al.^9^ and Kumar et al.^40^ for SOA from actual biomass-burning emissions. The light-absorption properties reported by those studies place their biomass-burning SOA within the M-BrC class. It is possible that this discrepancy can be partly because those studies performed photochemical aging in a smog chamber, where OH exposures are typically lower than those in the OFR. Because SOA is susceptible to photobleaching, shorter aging time scales may result in greater SOA absorption. Furthermore, the SOA in those studies was generated from the emissions of fine and woody fuels, which combust at higher temperatures than duff,^7^ potentially promoting pathways that are conducive for the formation of more light-absorbing SOA. However, we note that those studies retrieved the light-absorption properties of SOA indirectly from the light-absorption measurements of AOA and POA, assuming no change in POA absorption due to photochemical aging (i.e., similar to our *k*_SOA_ (from POA) approach). Therefore, as described in Section 3.2, SOA light absorption in those studies can be severely overestimated due to ignoring the photoenhancement of POA in the analysis.

### 3.4 Effect of Solvent Extraction on the Retrieval of Light-Absorption Properties of OA

We have previously demonstrated that relying on solvent extraction to retrieve aerosol light-absorption properties can lead to significant underestimation,^5,7,57^ with the degree of underestimation being dependent on the aerosol solubility in the chosen solvent. On the one hand, Cheng et al.^57^ demonstrated that *k* of nigrosin (which is water-soluble) retrieved using water extraction followed by UV–vis spectroscopy yielded results that were in close agreement with those retrieved from online measurements of the nigrosin aerosol (i.e., using optical closure). On the other hand, using methanol or water extraction for biomass-burning POA led to significant underestimation of *k* values compared to those retrieved from online measurements because biomass-burning POA absorption is mostly dictated by insoluble S-BrC.^5,7^ In this study, we evaluated how the bias in the retrieval of *k* using methanol extraction impacted the different OA components (POA, AOA, APOA, and SOA).

Figure 5 shows that methanol extracts of POA, APOA, and AOA consistently exhibited smaller *k* values (Figure 5a) and larger *w* (Figure 5b) compared to their aerosol counterparts, suggesting that the BrC in these three OA components consisted of species that were insoluble in methanol but contributed significantly to light absorption. For example, the *k*_POA,550_ (aerosol) is 2.3 times larger than its *k*_POA,550_ (methanol extracts) while its *w*_POA_ (aerosol) is smaller by a factor of 1.6 than *w*_POA_ (methanol extracts). These discrepancies are qualitatively consistent with our previous works that showed that methanol extracts and water extracts of biomass-burning POA were significantly less absorbing than the aerosol.^5,7^ In contrast, SOA exhibited very good agreement between the light-absorption properties of the aerosol and methanol extracts. Specifically, the *k*_SOA,550_ and *w*_SOA_ values of the aerosol and methanol extracts were within 2% and 6%, respectively. This close agreement indicates that, unlike POA, SOA does not include insoluble components with appreciable absorption.

**Figure 5:**
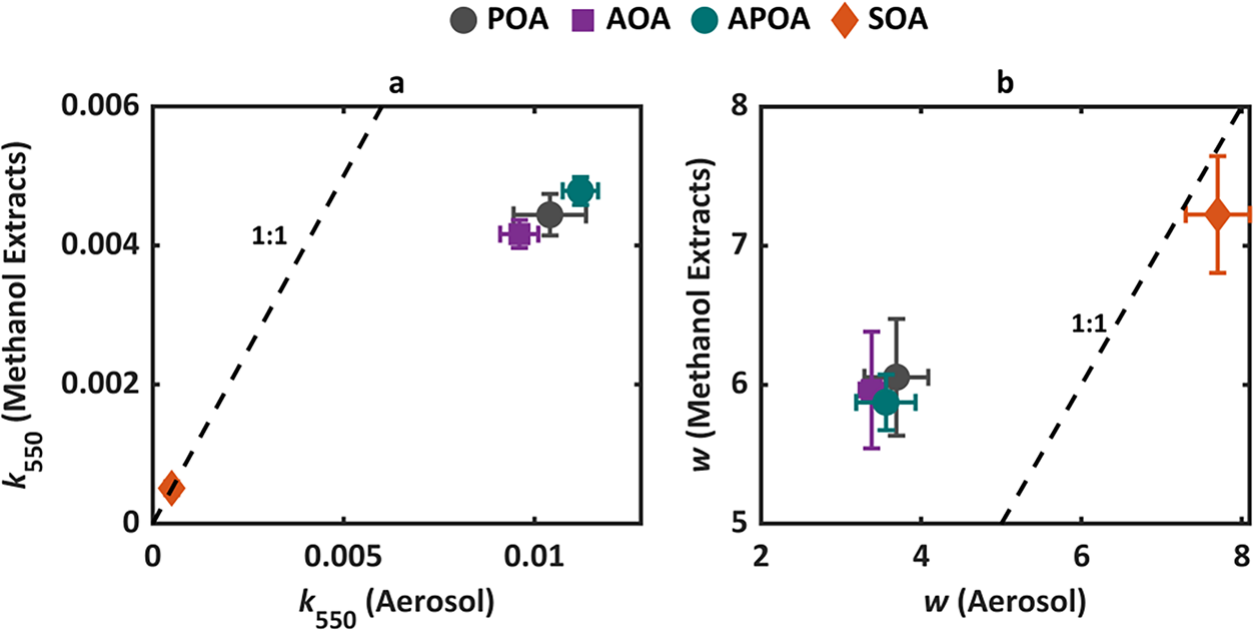
Comparison of light-absorption properties of organic aerosol (OA) retrieved from online photoacoustic measurements (aerosol) and offline UV–vis measurements of methanol-soluble extracts. (a) Imaginary part of the refractive index at 550 nm (*k*_550_); (b) wavelength dependence of *k* (*w*). Measurements for AOA, APOA, and SOA correspond to 0.5-day equivalent atmospheric photochemical aging. Error bars reflect standard deviations. Numerical values are shown in Table S4.

Overall, these results illustrate that from the perspective of quantifying light-absorption properties, methanol extraction produces accurate results for SOA, indicating that its chromophores are mostly associated with methanol-soluble (likely polar) species. However, methanol extraction would severely underestimate POA absorption, indicating that its dominant chromophores are associated with larger molecular size and methanol-insoluble species (likely nonpolar).^4^ Furthermore, photochemical aging of POA does not render its dominant chromophores more methanol-soluble, as illustrated by the persistent discrepancy between light-absorption properties of aerosol and methanol extracts for APOA and AOA.

### 3.5 Evolution of the Chemical Composition of OA Due to Photochemical Aging

In this section, we investigate whether and how the evolution of light-absorption properties of POA and SOA associated with photochemical aging (Section 3.2) is reflected in their respective chemical composition. To that end, we utilized van Krevelen plots to compare the molecular assignments of APOA at different equivalent atmospheric photochemical ages to those of POA. We also compared the molecular assignments of SOA at 1 day, 1.5 days, and 2 days to those of SOA at 0.5 days. As demonstrated by the gray points in Figure 6, POA exhibited relatively modest compositional changes during photochemical aging, with 76%, 75%, 74%, and 64% of its initial molecular formulas retained at 0.5 days, 1 day, 1.5 days, and 2 days, respectively. This molecular persistence highlights the relative chemical stability of the molecular constituents of POA emitted from duff burning. In sharp contrast, the SOA exhibited a substantial chemical change during photochemical aging. Figure 7 shows that, relative to SOA at 0.5 days, only 24%, 19%, and 10% of the molecules persisted to 1 day, 1.5 days, and 2 days, respectively. This indicates rapid transformation of SOA molecules, likely driven by oxidative fragmentation into smaller, more saturated species.^6,33^

**Figure 6:**
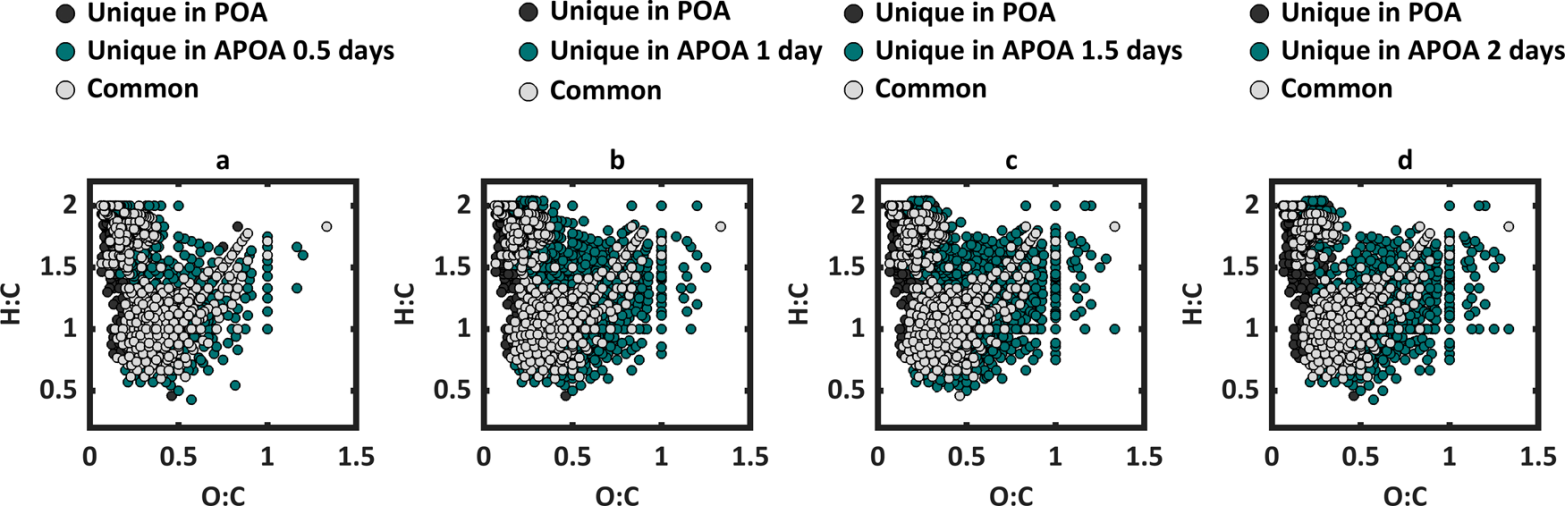
Van Krevelen plots comparing molecular assignments detected using ESI-FTICR-MS of POA to APOA at (a) 0.5 days of equivalent photochemical aging, (b) 1 day of equivalent photochemical aging, (c) 1.5 days of equivalent photochemical aging, and (d) 2 days of equivalent photochemical aging, The gray dots correspond to molecules common between the POA and APOA, and the other color dots correspond to molecules unique to each.

**Figure 7:**
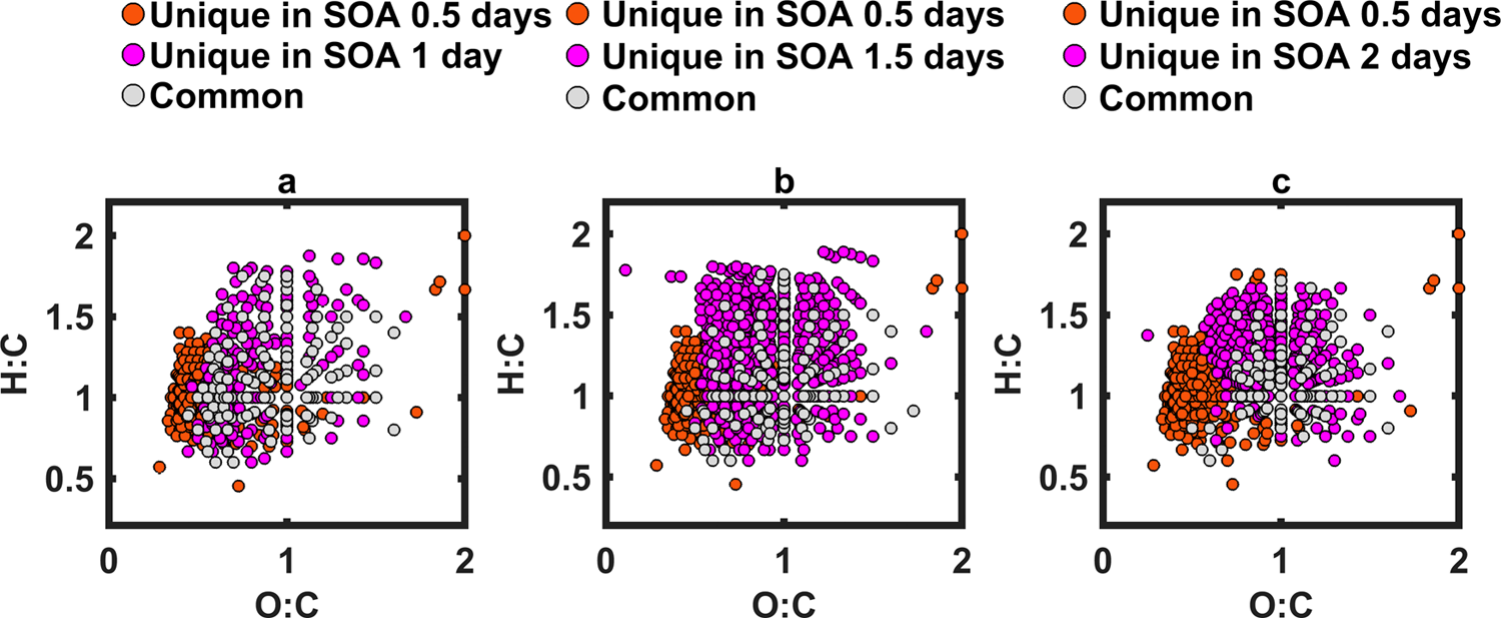
Van Krevelen plots comparing molecular assignments detected using ESI-FTICR-MS of SOA at (a) 0.5 days and 1 day of equivalent photochemical aging, (b) 0.5 days and 1.5 days of equivalent photochemical aging, and (c) 0.5 days and 2 days of equivalent photochemical aging.

The gray dots correspond to molecules common between the two OA types, and the other color dots correspond to molecules unique to each OA type.

The contrasting evolution in chemical composition of POA and SOA due to photochemical aging is further illustrated by modified aromaticity index (AI_mod_)-based molecular classification (see SI for details).^88–90^ As shown in Figure 8, APOA maintained relatively consistent distributions of AI_mod_ classes, with only a slight increase in the high O unsaturated fraction. SOA, however, exhibited a sharp decline in both high O and low O unsaturated species over the aging time, coinciding with the loss of its absorption (Section 3.2). The high O unsaturated class–defined by moderate aromaticity and high oxygen content–emerges as a key distinguishing feature between the two OA types. Its depletion in SOA and progressive increase in APOA with aging correlate with a decrease in SOA absorption and an increase in APOA absorption, respectively (Section 3.2), suggesting that these species potentially constitute a significant portion of the methanol-soluble BrC chromophores in duff-burning POA and SOA.

**Figure 8:**
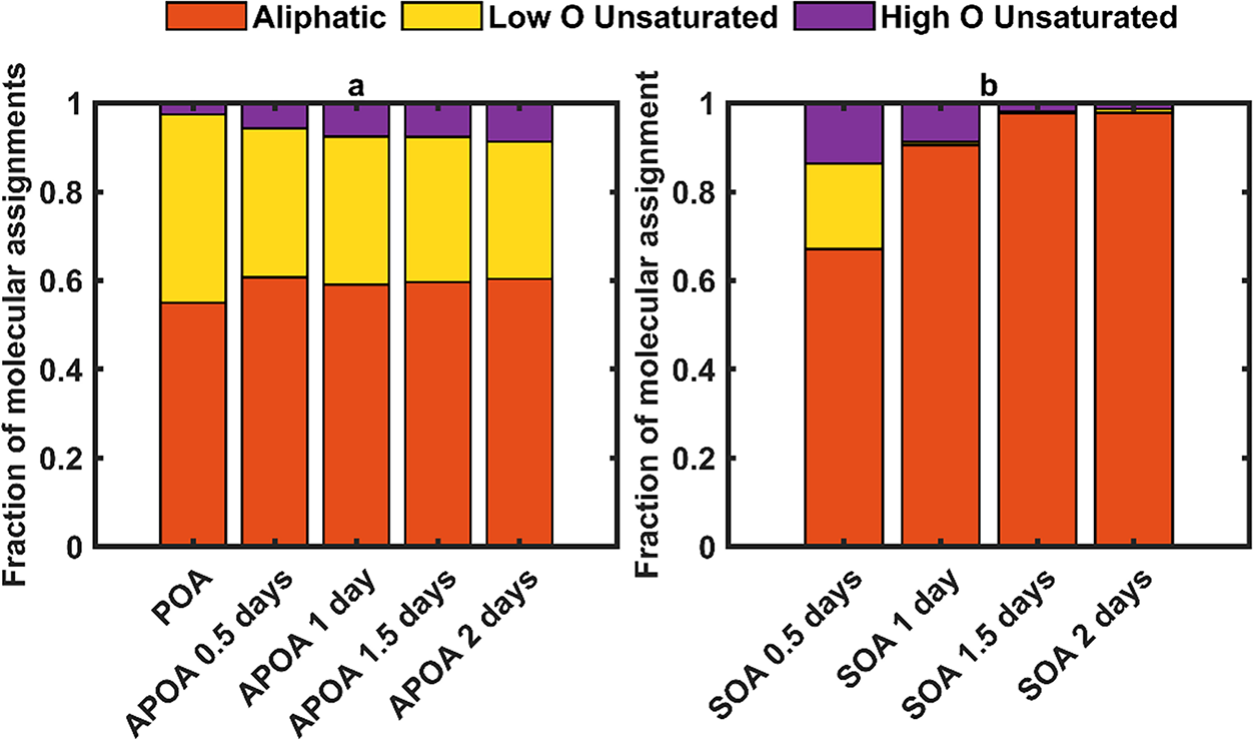
Evolution of molecular classes during photochemical aging, classified using the modified aromaticity index (AI_mod_) and oxygen-to-carbon ratio (O:C) for (a) POA, and (b) SOA. Bars represent the fractional abundance of four classes:^90^ aliphatic (AI_mod_ < 0), low O unsaturated (0 < AI_mod_ ≤ 0.5, O:C < 0.5), high O unsaturated (0 < AI_mod_ ≤ 0.5, O:C ≥ 0.5), and aromatic (AI_mod_ > 0.5). More detailed calculations for each class are shown in the SI.

We note that the chemical analysis presented here are for species that are methanol-soluble and efficiently ionized with ESI. The fact that the light-absorption properties of methanol-soluble SOA were in good agreement with those of SOA aerosol (Section 3.4) suggests that SOA was largely methanol-soluble, and it is likely that the chemical analysis of SOA is representative of the SOA itself. However, the chemical analysis of POA and APOA misses the methanol-insoluble species that are responsible for most of its absorption (Section 3.4). Nevertheless, the findings apply at least to the methanol-soluble fraction of the POA and indicate highly contrasting behaviors of SOA and POA: SOA is highly susceptible to oxidative transformation, undergoing rapid molecular evolution and loss of chromophoric functionality during photochemical aging, whereas POA is comparatively resistant to such transformation, preserving a stable core of light-absorbing species.

## 4 Conclusion

This study provides the first direct quantification of the light-absorption properties of SOA formed from actual biomass-burning emissions under controlled photochemical aging. By isolating POA and SOA from duff-burning emissions and aging them separately in an OFR, we resolved their distinct optical evolution. Our findings demonstrate that SOA derived from duff-burning emissions is very weakly absorbing and undergoes rapid photobleaching with increased photochemical aging, rendering it virtually nonabsorbing after 2 days of equivalent atmospheric aging. In contrast, POA exhibits an increase in absorption over the 2 days of aging, indicating a photoenhancement effect. These distinct aging behaviors result in a net decrease in the AOA absorption, not due to the photobleaching of the POA, but rather due to the formation and subsequent photobleaching of very weakly absorbing SOA. Chemical composition analysis corroborated the distinct evolution of light-absorption properties of POA and SOA, revealing a modest transformation in POA and an extensive transformation in SOA. Moreover, the distinct evolution of the light-absorption properties of SOA and POA is in part attributable to the loss of unsaturated oxygenated species in SOA and their contrasting increase in POA. These results underscore the importance of decoupling SOA and POA when assessing the optical evolution of biomass-burning aerosols during atmospheric aging.

Furthermore, we probed previous approaches that estimated SOA absorption by subtracting POA absorption from the absorption of AOA. We demonstrated that such assumptions can overestimate SOA absorption by close to an order of magnitude, due to the neglect of aging-induced evolution in POA absorption. Instead, the use of APOA light-absorption properties provided significantly more accurate estimates of SOA absorption. Therefore, when using an indirect approach to estimate SOA absorption, there is a need to account for the optical evolution of POA during photochemical aging.

Overall, these findings highlight the transient nature of duff-burning SOA and its limited contribution to atmospheric BrC absorption. If the findings from this study translate to other biomass-burning SOA systems, they would indicate that, to the first order, biomass-burning SOA can be assumed to be nonabsorbing in radiative-transfer calculations. The challenge in accurately quantifying the effect of photochemical aging on biomass-burning OA absorption lies in the uncertainty in quantifying the amount of SOA formation rather than its light-absorption properties.

## Supplementary Material



## Notes

Calculations for the imaginary refractive index of methanol extracts, OCEC data tables, and data on light absorption properties used in Figures 4 and 5 (PDF)
