# Supplementary material for: Decoupling the Evolution of the Light-Absorption Properties of Primary and Secondary Organic Aerosol Produced from Duff Burning
Source: ACS EST Air. 2025 Nov 21;2(12):2966–78. doi: 10.1021/acsestair.5c00274 (PMC12706742; doi:10.1021/acsestair.5c00274)
Supplement: Supplementary file 1 [file ea5c00274_si_001.pdf]

Supporting information for:

Decoupling the Evolution of the Light-absorption Properties of Primary and Secondary Organic Aerosol Produced from Duff Burning

Muhammad I. Abdurrahman[1], Chase K. Glenn[3], Robert Penland[1], Ryan P. Poland[2], Jonathan H. Choi [2], Elijah T. Roberts[2], I. Jonathan Amster[2], Geoffrey D. Smith[2], Rawad Saleh[1]*

[1]School of Environmental, Civil, Agricultural and Mechanical Engineering, University of Georgia, Athens, GA, 30602, USA.

[2]Department of Chemistry, University of Georgia, Athens, GA 30602, USA.

[3]Aerodyne Research Inc., Billerica, MA 01821, USA.

*Email: rawad@uga.edu

S1. Calculations for the imaginary part of the refractive index (*k*) of methanol extracts

The *k* of methanol extracts was calculated based on UV–vis spectrophotometric absorbance measurements. The procedure involves converting the measured absorbance (A(λ)) at each wavelength λ to the absorption coefficient (α), and then to *k*(λ) using[1]:

$$\alpha = \frac{\ln(10)\ \mathrm{A}(\lambda)}{\mathrm{C}\times\mathrm{L}} \qquad \text{(S1)}$$

$$k(\lambda) = \frac{\lambda \times \alpha}{4 \times \pi} \tag{S2}$$

Where A(λ) is the absorbance at wavelength λ, C is the concentration of organic carbon in solution, and L is the optical path length of the cuvette (typically 1 cm).

The organic mass concentration (C) of each extract was determined using the following expression:

$$C = \frac{\mathrm{OC} \times 1.8}{V_p} \tag{S3}$$

Where OC is the mass of organic carbon on the filter punch, measured via OCEC analysis, and V_p is the volume of methanol extract solution deposited onto the punch. The factor 1.8 accounts for the typical carbon-to-organic matter conversion factor for biomass burning aerosols.[1]

S2. Chemical classification OA using modified aromaticity index

The modified aromaticity index (AI_{mod}) was calculated using molecular formulas derived from electrospray ionization Fourier-transform ion cyclotron resonance mass spectrometry (ESI-FTICR-MS), following the method outlined by Koch and Dittmar.[2,3] The AI_{mod} is given by:

$$\mathrm{AI_{mod}} = \frac{1 + \mathrm{C} - \frac{1}{2}\,\mathrm{O} - \mathrm{S} - \frac{1}{2}\,\mathrm{H} - \frac{1}{2}\,\mathrm{N}}{\mathrm{C} - \frac{1}{2}\,\mathrm{O} - \mathrm{S} - \mathrm{N}} \tag{S4}$$

where C, H, O, S, and N represent the number of carbon, hydrogen, oxygen, sulfur, and nitrogen atoms, respectively. In our analysis, the OA molecular formulas were limited to compounds containing only carbon, hydrogen, oxygen, and nitrogen. Molecules were categorized according to Vandergrift et al.[4] based on their AI_{mod} values:

- **Aliphatic:** AImod < 0
- **Low-oxygen unsaturated:** $0 < AI_{mod} \leq 0.5$ and $O/C < 0.5$

- **High-oxygen unsaturated:** $0 < AI_{mod} \leq 0.5$ and $O/C \geq 0.5$
- **Aromatic:** $AI_{mod} > 0.5$

Tables

Table S1. Relative amounts of organic carbon (OC) and elemental carbon (EC) measured using the OCEC analyzer for the aged organic aerosol (AOA) at different photochemical aging times.

**Sample ID**	**OC (µg/cm²)**	**EC (µg/cm²)**	**TC (µg/cm²)**	**EC/TC ratio**
Blank	0.9210 ± 0.1461	0.0010 ± 0.1001	0.9220 ± 0.2461	0.0011
AOA 0.5 days	40.7030 ± 2.5351	0.0349 ± 0.1000	40.7379 ± 2.6352	0.0009
AOA 1 day	23.7048 ± 1.6852	0.0080 ± 0.1000	23.7128 ± 1.7853	0.0003
AOA 1.5 days	67.4450 ± 3.8722	0.0090 ± 0.1101	67.4540 ± 3.9824	0.0001
AOA 2 days	93.4740 ± 4.7737	0.0196 ± 0.1859	93.4936 ± 4.9596	0.0002

Table S2. Relative amounts of organic carbon (OC) and elemental carbon (EC) measured using the OCEC analyzer for the primary organic aerosol (POA) and aged primary organic aerosol (APOA) at different photochemical aging times.

**Sample ID**	**OC (µg/cm²)**	**EC (µg/cm²)**	**TC (µg/cm²)**	**EC/TC ratio**
Blank	0.3715 ± 0.1186	0.0004 ± 0.1001	0.3718 ± 0.2186	0.0009
POA	49.0524 ± 2.6526	0.2249 ± 0.1315	49.2774 ± 2.7841	0.0046
APOA 0.5 days	56.2518 ± 3.0126	0.2463 ± 0.1345	56.4981 ± 3.1471	0.0044
APOA 1 day	70.6117 ± 3.7306	0.2840 ± 0.1398	70.8957 ± 3.8703	0.0040
APOA 1.5 days	54.2229 ± 2.9111	0.2024 ± 0.1283	54.4253 ± 3.0395	0.0037

APOA 2 days	48.7462 ± 2.6373	0.1058 ± 0.1148	48.8521 ± 2.7521	0.0022

Table S3. Numerical values used for Figure 4 (optical classification) in the main text. Values include k_{550} and *w,* measured in this study and those reported in the literature. Literature values that were not reported as k_{550} and *w* were converted using the procedure described in Saleh[5].

Sample	k_{550}	*w*
POA	0.00992 ± 0.00095	3.63 ± 0.36
AOA 0.5 days	0.00960 ± 0.00050	3.38 ± 0.15
SOA 0.5 days	0.00050 ± 0.00010	7.71 ± 0.66
APOA 0.5 days	0.01120 ± 0.00046	3.55 ± 0.37
SOA (from POA) 0.5 days	0.00533	4.95
SOA (from APOA) 0.5 days	0.00081	8.96
Lambe et al.[6]	0.00038	8.00
Liu et al.[7]	0.00015	7.90
Joo et al.[8]	0.00257	6.00
Jiang et al.[9]	0.00269	6.03
Yang et al.[10]	0.00044	5.42
Saleh et al.[11]	0.01725 ± 0.00009	4.65 ± 1.13
Kumar et al.[12]	0.02200	4.60
Glenn et al.[13]	0.00400 ± 0.00100	3.44 ± 0.42

Table S4. Numerical values for *k* of aerosol and methanol extracts used in Figure 5 in the main text.

Sample	k_{550} (aerosol)	k_{550} (methanol extract)	*w* (aerosol)	*w* (methanol extract)
POA	0.0104 ± 0.00095	0.00444 ± 0.0003	3.69 ± 0.40	6.05 ± 0.42
AOA 0.5 days	0.0096 ± 0.0005	0.00417 ± 0.0002	3.38 ± 0.15	5.96 ± 0.42
APOA 0.5 days	0.0112 ± 0.00046	0.00479 ± 0.0002	3.56 ± 0.37	5.87 ± 0.20
SOA 0.5 days	0.0005 ± 0.0001	0.00051 ± 0.0001	7.70 ± 0.40	7.22 ± 0.42

References

(1) Atwi, K.; Cheng, Z.; El Hajj, O.; Perrie, C.; Saleh, R. A Dominant Contribution to Light Absorption by Methanol-Insoluble Brown Carbon Produced in the Combustion of Biomass Fuels Typically Consumed in Wildland Fires in the United States. *Environ. Sci. Atmospheres* **2022**, *2* (2), 182–191. https://doi.org/10.1039/D1EA00065A.

(2) Koch, B. P.; Dittmar, T. From Mass to Structure: An Aromaticity Index for High-resolution Mass Data of Natural Organic Matter. *Rapid Commun. Mass Spectrom.* **2006**, *20* (5), 926–932. https://doi.org/10.1002/rcm.2386.

(3) Koch, B. P.; Dittmar, T. From Mass to Structure: An Aromaticity Index for High-Resolution Mass Data of Natural Organic Matter. *Rapid Commun. Mass Spectrom.* **2016**, *30* (1), 250–250. https://doi.org/10.1002/rcm.7433.

(4) Vandergrift, G. W.; Shawon, A. S. M.; Dexheimer, D. N.; Zawadowicz, M. A.; Mei, F.; China, S. Molecular Characterization of Organosulfate-Dominated Aerosols over Agricultural Fields from the Southern Great Plains by High-Resolution Mass Spectrometry. *ACS Earth Space Chem.* **2022**, *6* (7), 1733–1741. https://doi.org/10.1021/acsearthspacechem.2c00043.

(5) Saleh, R. From Measurements to Models: Toward Accurate Representation of Brown Carbon in Climate Calculations. *Curr. Pollut. Rep.* **2020**, *6* (2), 90–104. https://doi.org/10.1007/s40726-020-00139-3.

(6) Lambe, A. T.; Cappa, C. D.; Massoli, P.; Onasch, T. B.; Forestieri, S. D.; Martin, A. T.; Cummings, M. J.; Croasdale, D. R.; Brune, W. H.; Worsnop, D. R.; Davidovits, P. Relationship between Oxidation Level and Optical Properties of Secondary Organic

Aerosol. *Environ. Sci. Technol.* **2013**, *47* (12), 6349–6357. https://doi.org/10.1021/es401043j.

(7) Liu, P. F.; Abdelmalki, N.; Hung, H.-M.; Wang, Y.; Brune, W. H.; Martin, S. T. ACP - Ultraviolet and Visible Complex Refractive Indices of Secondary Organic Material Produced by Photooxidation of the Aromatic Compounds Toluene and m-Xylene. **2015**.

(8) Joo, T.; Machesky, J. E.; Zeng, L.; Hass-Mitchell, T.; Weber, R. J.; Gentner, D. R.; Ng, N. L. Secondary Brown Carbon Formation From Photooxidation of Furans From Biomass Burning. *Geophys. Res. Lett.* **2024**, *51* (1), e2023GL104900. https://doi.org/10.1029/2023GL104900.

(9) Jiang, F.; Siemens, K.; Linke, C.; Li, Y.; Gong, Y.; Leisner, T.; Laskin, A.; Saathoff, H. Molecular Analysis of Secondary Organic Aerosol and Brown Carbon from the Oxidation of Indole. *Atmospheric Chem. Phys.* **2024**, *24* (4), 2639–2649. https://doi.org/10.5194/acp-24-2639-2024.

(10) Yang, Z.; Tsona, N. T.; George, C.; Du, L. Nitrogen-Containing Compounds Enhance Light Absorption of Aromatic-Derived Brown Carbon. *Environ. Sci. Technol.* **2022**, *56* (7), 4005–4016. https://doi.org/10.1021/acs.est.1c08794.

(11) Saleh, R.; Hennigan, C. J.; McMeeking, G. R.; Chuang, W. K.; Robinson, E. S.; Coe, H.; Donahue, N. M.; Robinson, A. L. Absorptivity of Brown Carbon in Fresh and Photo-Chemically Aged Biomass-Burning Emissions. *Atmospheric Chem. Phys.* **2013**, *13* (15), 7683–7693. https://doi.org/10.5194/acp-13-7683-2013.

(12) Kumar, N. K.; Corbin, J. C.; Bruns, E. A.; Massabó, D.; Slowik, J. G.; Drinovec, L.; Močnik, G.; Prati, P.; Vlachou, A.; Baltensperger, U.; Gysel, M.; El-Haddad, I.; Prévôt, A. S. H. Production of Particulate Brown Carbon during Atmospheric Aging of Residential Wood-Burning Emissions. **2018**.

(13) Glenn, C. K.; El Hajj, O.; McQueen, Z.; Poland, R. P.; Penland, R.; Roberts, E. T.; Choi, J. H.; Bai, B.; Shin, N.; Anosike, A.; Kumar, K. V.; Abdurrahman, M. I.; Liu, P.; Amster, I. J.; Smith, G. D.; Flanagan, S.; Callaham, M. A.; Loudermilk, E. L.; O'Brien, J. J.; Saleh, R. Brown Carbon Emissions from Biomass Burning under Simulated Wildfire and Prescribed-Fire Conditions. *ACS EST Air* **2024**, *1* (9), 1124–1136. https://doi.org/10.1021/acsestair.4c00089.
